# Evolving Patterns of Initial RRMS Treatment in Finland (2013–2022): Insights From a Nationwide Multiple Sclerosis Register

**DOI:** 10.1002/brb3.70326

**Published:** 2025-02-11

**Authors:** Henrik Ahvenjärvi, Elina Jokinen, Matias Viitala, Henri Autio, Anne M. Portaankorva, Merja Soilu‐Hänninen, Johanna Krüger, Mervi Ryytty

**Affiliations:** ^1^ Research Unit of Clinical Medicine, Neurology University of Oulu Oulu Finland; ^2^ Novartis Finland Ltd Espoo Finland; ^3^ StellarQ Ltd Turku Finland; ^4^ Medical Research Center Oulu University Hospital Oulu Finland; ^5^ Clinical Neurosciences University of Helsinki Helsinki Finland; ^6^ Clinical Neurosciences University of Turku Turku Finland; ^7^ Neurocenter Turku University Hospital Turku Finland; ^8^ Neurocenter, Neurology Oulu University Hospital Oulu Finland

**Keywords:** COVID‐19, delayed diagnosis, drug therapy, multiple sclerosis, routinely collected health data

## Abstract

**Background:**

The treatment of relapsing‐remitting multiple sclerosis (RRMS) is changing. There are limited data about initial treatment of RRMS in Finland.

**Objective:**

Our objectives were to study the trends of initial disease‐modifying treatments (DMTs) for patients with RRMS from 2013 to 2022, treatment delays, factors associated with DMT choice, DMT switch patterns, and the effect of the COVID‐19 pandemic.

**Methods:**

This retrospective register study used secondary data from the Finnish MS register. The DMTs were classified into medium‐efficacy DMTs (meDMTs; beta interferons, glatiramer acetate, fumarates, and teriflunomide) and high‐efficacy DMTs (heDMTs; alemtuzumab, cladribine, daclizumab, natalizumab, ocrelizumab, ofatumumab, and rituximab).

**Results:**

The inclusion criteria were fulfilled by 2479 individuals. From 2013 to 2022, the proportion of heDMTs as the initial therapy increased by 5.3‐fold from 6.9% to 43.7% (*p* < 0.001). Median diagnostic delay decreased from 10.1 to 4.6 months (*p* < 0.001). The COVID‐19 pandemic did not cause treatment delays. Higher disease activity and younger age were associated with the choice of heDMT as the initial DMT. heDMTs were the preferred second DMT in patients switching due to lack of efficacy.

**Conclusion:**

In Finland, the treatment of RRMS has shifted toward earlier diagnosis and earlier initiation of heDMTs, likely improving the prognosis of the patients.

## Graphical abstract

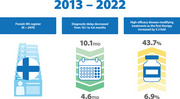

## 1 Introduction

Revolutionary advancements have been made in the treatment of multiple sclerosis (MS) over the past decade due to improved diagnostic criteria, novel disease‐modifying therapies (DMTs), and new insights on treatment strategies. Recent trends in initial RRMS treatment and diagnostics in Finland have not been studied before. Previous register studies conducted in other countries suggest heterogeneity in treatment strategies (Hrnciarova et al. 2023; Spelman et al. 2021) and a recent decrease in diagnostic delays (Magyari et al. 2022; Blaschke et al. 2022; Swedish MS Registry [SMSREG] 2024). Limited information is available regarding the most used DMTs following the initial DMTs. To our knowledge, no previous studies have assessed the impact of the COVID‐19 pandemic on treatment delays in MS.

Our primary objective was to investigate the trends in the choice of initial DMTs for patients with RRMS between the years 2013 and 2022 in Finland. The secondary objectives were as follows: to evaluate how the time from the first presentation of symptoms to the diagnosis of RRMS and to the initiation of the first DMT has evolved between 2013 and 2022, to review the factors governing the choice of the initial DMTs, to investigate the reasons for the discontinuation of the first DMT, to describe the most common DMT switch patterns for the first and the second switch, to understand the nature of the switches in terms of efficacy, and to study the effect of COVID‐19 outbreak on the treatment delays.

## 2 Materials and Methods

### 2.1 Study Design

This study used secondary data from the Finnish MS register (Laakso et al. 2019). Overall, data from 16 of 21 Finnish well‐being service counties were included in our study (about 85% of national coverage of patients with MS), Southwest Finland, Satakunta, Pirkanmaa, Päijät‐Häme, Kymenlaakso, South Karelia, North Savo, Central Finland, South Ostrobothnia, North Ostrobothnia, Ostrobothnia, Kainuu, East Uusimaa, Central Uusimaa, West Uusimaa, and Vantaa and Kerava.

**FIGURE 1:**
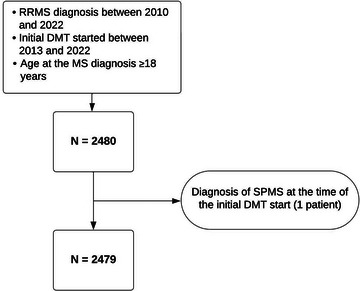
The inclusion process. DMT, disease‐modifying therapy; MS, multiple sclerosis; RRMS, relapsing‐remitting multiple sclerosis; SPMS, secondary progressive multiple sclerosis.

The inclusion criteria were an RRMS diagnosis between 2010 and 2022, initiation of initial DMT between 2013 and 2022, and age at diagnosis ≥ 18 years (Figure 1). The exclusion criterion was the diagnosis of secondary progressive MS (SPMS) at the initiation of the first DMT.

We used the STROBE cross‐sectional reporting guidelines (von Elm et al. 2007).

### 2.2 Variables

The following variables were studied: sex, age, time and the type of MS symptom onset, the time of diagnosis, Expanded Disability Status Scale (EDSS) (Kurtzke 1983) score at the time of DMT initiation, the history of relapses and DMT usage, and reasons for DMT stoppages.

The DMTs were classified as follows: (1) The medium efficacy injectables (meINJs) included interferon betas and glatiramer acetate. (2) The medium‐efficacy oral therapies (meORALs) encompassed dimethyl fumarate, diroximel fumarate, and teriflunomide. (3) The sphingosine 1‐phosphate receptor modulators (S1PRms) included fingolimod and ponesimod. (4) The heDMT category consisted of alemtuzumab, cladribine, daclizumab, natalizumab, ocrelizumab, ofatumumab, and rituximab (off‐label treatment). (5) Other therapies included azathioprine and mitoxantrone.

meINJs and meORALs were considered as meDMTs. Statistical group comparisons were made between meDMTs and heDMTs. A standardized efficacy classification of DMTs does not exist, and studies have classified S1PRms variably. In this study, S1PRms were not included in the efficacy group analyses due to their efficacy falling between heDMTs and meDMTs (Tramacere et al. 2015).

The symptoms at onset were categorized according to their clinical origin within the central nervous system (CNS) as follows: (1) brainstem (double vision, specific brain nerve, and other cranial nerve symptoms), (2) cerebellum (coordination and balance disturbances), (3) spinal cord (bladder and bowel dysfunction), (4) pyramidal tract (muscle weakness and spasticity), (5) optic nerve (optic neuritis), (6) sensory pathways (paresthesia and dysesthesia), and (7) unknown. Symptoms at onset were considered multifocal when ≥ 2 symptoms were present, excluding sensory and unknown symptoms, as their origin from a distinct CNS area cannot be reliably determined.

Time periods from 2013 to 2019 and from 2020 to 2022 were compared in the analyses due to two reasons. First, the COVID‐19 pandemic took place in Finland during the years 2020–2022. Second, the Finnish current care guidelines for MS (The Finnish Medical Society Duodecim and the Finnish Neurological Society 2020), including the most recent diagnostic criteria (Thompson et al. 2018), were updated in January 2020.

### 2.3 Statistical Analysis

Data analysis and visualization were performed on pseudonymized data using RStudio (Version 2021.09.1). Partial dates were imputed as the middle of the month or year. Baseline EDSS score was defined as the last recorded EDSS score within 1 year before the start of initial DMT, and annualized relapse rate (ARR) was defined as the number of relapses during that year.

Numerical variables were expressed as means with standard deviations (SDs) or medians with interquartile ranges (IQRs). Categorical variables were expressed as frequencies and proportions based on nonmissing data. Group comparisons were performed using nonparametric methods. Wilcoxon rank sum test was used for calculating *p* values for continuous variables and Fisher's exact test for categorical variables. Logistic regression model was fitted using generalized linear model with logit link function to get odds ratios and confidence intervals for heDMT chosen as the initial DMT over meDMT. Usage of the selected variables was confirmed using variance inflation factors, and underlying multicollinearity was assessed. Diagnostic tests were conducted to identify any potential influential outliers. Benjamini–Hochberg procedure (1995) was used for controlling and checking the false discovery rate and for correcting *p* values for multiple comparisons between demographic and clinical variables.

Trend analysis for heDMTs was performed using both the Cochran–Armitage trend test and the Mann–Kendall trend test. In all analyses, two‐tailed or corrected *p* values below 0.05 were considered statistically significant. StellarQ Ltd., Finland was responsible for data analysis and visualization.

### 2.4 Standard Protocol Approvals, Registrations, and Patient Consents

This study was approved (THL/4118/14.02.00/2022) by the Finnish Social and Health Data Permit Authority, Findata (www.findata.fi/en). According to the Finnish legislation, since only retrospective register data were involved in the study without contact with patients, ethics board review or patient consent was not required.

## 3 Results

### 3.1 Demographic Characteristics

The inclusion criteria were fulfilled by 2479 patients with RRMS (Figure 1). Of all the patients, 72.5% were females (Table 1). The mean age of MS onset was 32.7 years (SD = 9.39). Overall, 12.4 % of patients had missing data on the onset date.

**TABLE 1:** The demographics of patients with RRMS receiving their initial DMT between 2013 and 2022 in Finland.

	All patients^a^ (*N* = 2479)	meINJs + meORALs (*n* = 1947)	heDMTs (*n* = 480)	S1PRms (*n* = 46)	*p* value^b^, ^c^
Sex					
Female; *n* (%)	1797 (72.5%)	1423 (73.1%)	339 (70.6%)	32 (67.3%)	0.31
Age variables (years); mean (SD)					
Age at MS onset	32.7 (9.39)	33.0 (9.51)	31.6 (8.90)	31.2 (8.32)	0.007^**^
Age at MS diagnosis	35.6 (9.61)	36.1 (9.64)	33.8 (9.27)	33.6 (8.37)	< 0.001^***^
Age at the start of the initial DMT	36.0 (9.62)	36.5 (9.63)	34.2 (9.29)	34.0 (8.42)	< 0.001^***^
Time delay variables (months); median [IQR]					
Time since MS onset to MS diagnosis	9.2 [3.7–36.5]	10.6 [4.1–41.5]	5.5 [2.0–19.0]	6.3 [3.1–24.0]	< 0.001^***^
Time since MS onset to the start of the initial DMT	13.0 [5.8–43.8]	14.4 [6.0–51.4]	8.4 [4.1–23.3]	12.2 [6.4–31.9]	< 0.001^***^
Time since MS diagnosis to the start of the initial DMT	1.9 [1.1–3.3]	1.9 [1.1–3.3]	2.0 [1.1–3.1]	2.7 [2.0–4.8]	0.81
Relapse variables; mean (SD)					
ARR 1 year before MS diagnosis	1.0 (0.73)	0.9 (0.70)	1.1 (0.83)	1.2 (0.69)	< 0.001^***^
ARR 1 year before the start of the initial DMT	1.0 (0.78)	0.9 (0.75)	1.2 (0.86)	1.3 (0.81)	< 0.001^***^
EDSS score at the start of the initial DMT (0–10); median [IQR]	1.5 [1.0–2.0]	1.5 [0.0–2.0]	2.0 [1.0–3.0]	1.5 [0.8–2.0]	< 0.001^***^
Patients on initial DMT at least 2 years^d^; *n* (%)	1269 (60.0%)	989 (57.2%)	245 (72.5%)	33 (78.6%)	< 0.001^***^
Onset symptom level; *n* (%)					
Brainstem	377 (18.0%)	291 (17.6%)	79 (19.9%)	6 (15.0%)	0.33
Cerebellum	375 (17.9%)	276 (16.7%)	92 (23.2%)	6 (15.0%)	0.005^***^
Optic nerve	501 (23.9%)	436 (26.4%)	54 (13.6%)	10 (25.0%)	< 0.001^***^
Pyramidal tract	353 (16.9%)	248 (15.0%)	98 (24.7%)	6 (15.0%)	< 0.001^***^
Sensory pathways	999 (47.7%)	775 (46.9%)	204 (51.4%)	18 (45.0%)	0.14
Spinal cord	56 (2.7%)	35 (2.1%)	19 (4.8%)	1 (2.5%)	0.007^**^
Unknown	386 (15.6%)	296 (15.2%)	83 (17.3%)	6 (13.0%)	—
Multifocal symptoms: yes; *n* (%)	187 (8.9%)	132 (8.0%)	51 (12.8%)	3 (7.5%)	0.005^**^

*Note*: meINJs (beta interferons and glatiramer acetate), meORALs (dimethyl fumarate, diroximel fumarate, and teriflunomide), heDMTs (alemtuzumab, cladribine, daclizumab, natalizumab, ocrelizumab, ofatumumab, and rituximab), S1PRms (fingolimod and ponesimod). Brainstem (double vision, specific brain nerve symptoms, and other cranial nerve symptoms), cerebellum (disturbances of coordination and ataxia, vertigo, and disturbances of balance and posture), optic nerve (optic neuritis), pyramidal tract (muscle weakness and spasticity), sensory pathways (paresthesia/dysesthesia), and spinal cord (bladder and bowel dysfunction).

Abbreviations: ARR, annualized relapse rate; DMT, disease‐modifying therapy; EDSS, Expanded Disability Status Scale; heDMTs, high‐efficacy disease‐modifying therapies; IQR, interquartile range, meINJs, medium‐efficacy injectable; meORALs, medium‐efficacy oral; MS, multiple sclerosis; S1PRms, sphingosine 1‐phosphate receptor modulators.

^a^ Includes all patients with initial DMT initiation between 2013 and 2022.

^b^ *p* values are calculated comparing heDMT patients to DMT groups meINJ and meORAL.

^c^ *p* values are corrected using Benjamini–Hochberg procedure to control the false discovery rate.

^d^ Patients with initial DMT initiation between 2021 and 2022 were excluded.

** *p* < 0.01.

*** *p* < 0.001.

### 3.2 Initial DMT Selection and Treatment Delays

#### 3.2.1 Trends in Initial DMTs From 2013 to 2022

**FIGURE 2:**
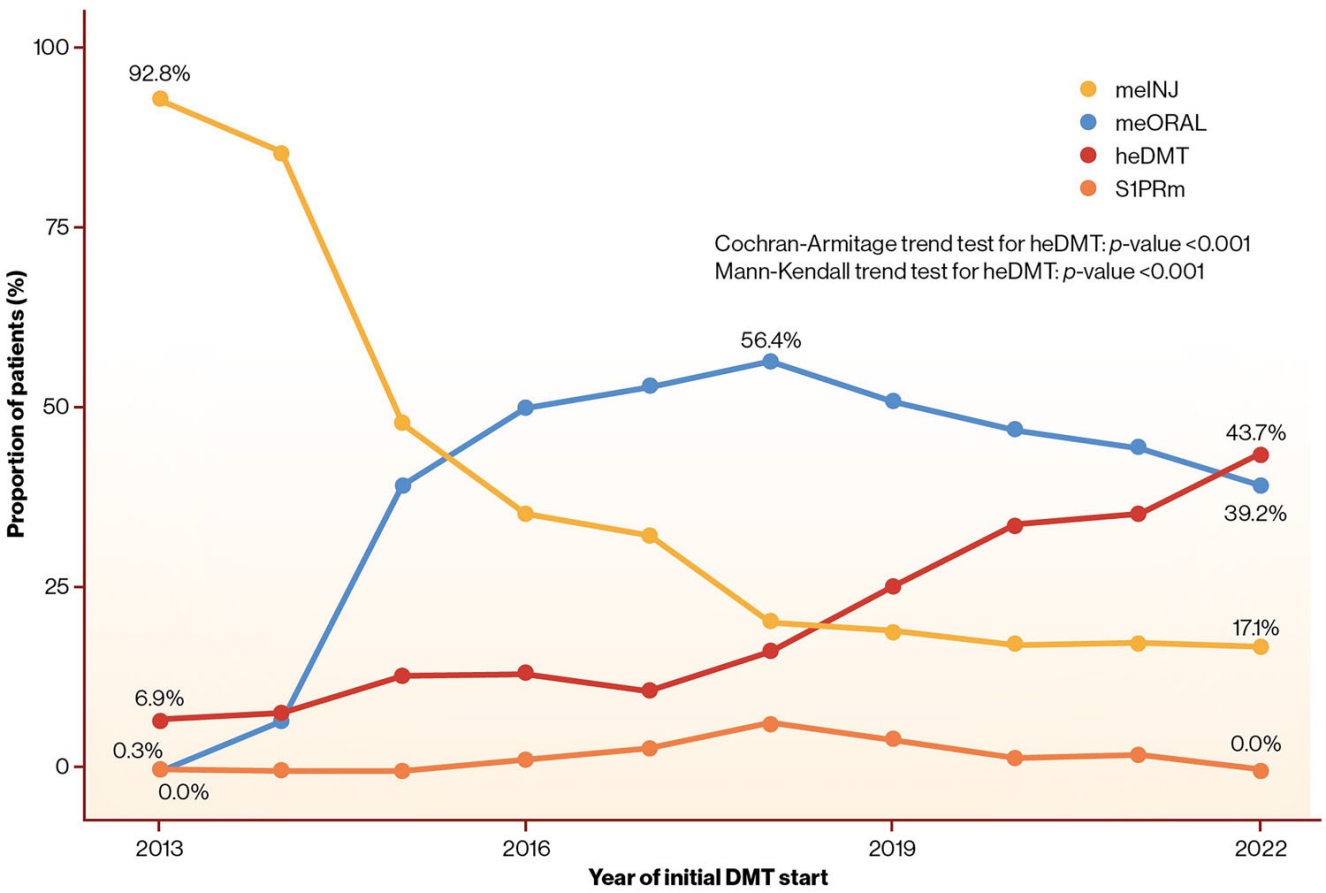
Evolution of the initial DMTs in Finland from 2013 to 2022. DMT, disease‐modifying therapy; heDMT, high‐efficacy disease‐modifying therapy; meINJ, medium‐efficacy injectable; meORAL, medium‐efficacy oral; S1PRm, sphingosine 1‐phosphate receptor modulator.

The percentage of heDMTs as the initial treatment increased from 6.9% in 2013 to 43.7% in 2022 (*p* < 0.001; Figure 2). Meanwhile, the proportion of meDMTs decreased from 92.8% in 2013 to 56.3% in 2022. The proportion of S1PRms remained low (maximum of 6.6% in 2018) during the whole observation period. The use of other DMTs was rare.

#### 3.2.2 Treatment Delays

**FIGURE 3:**
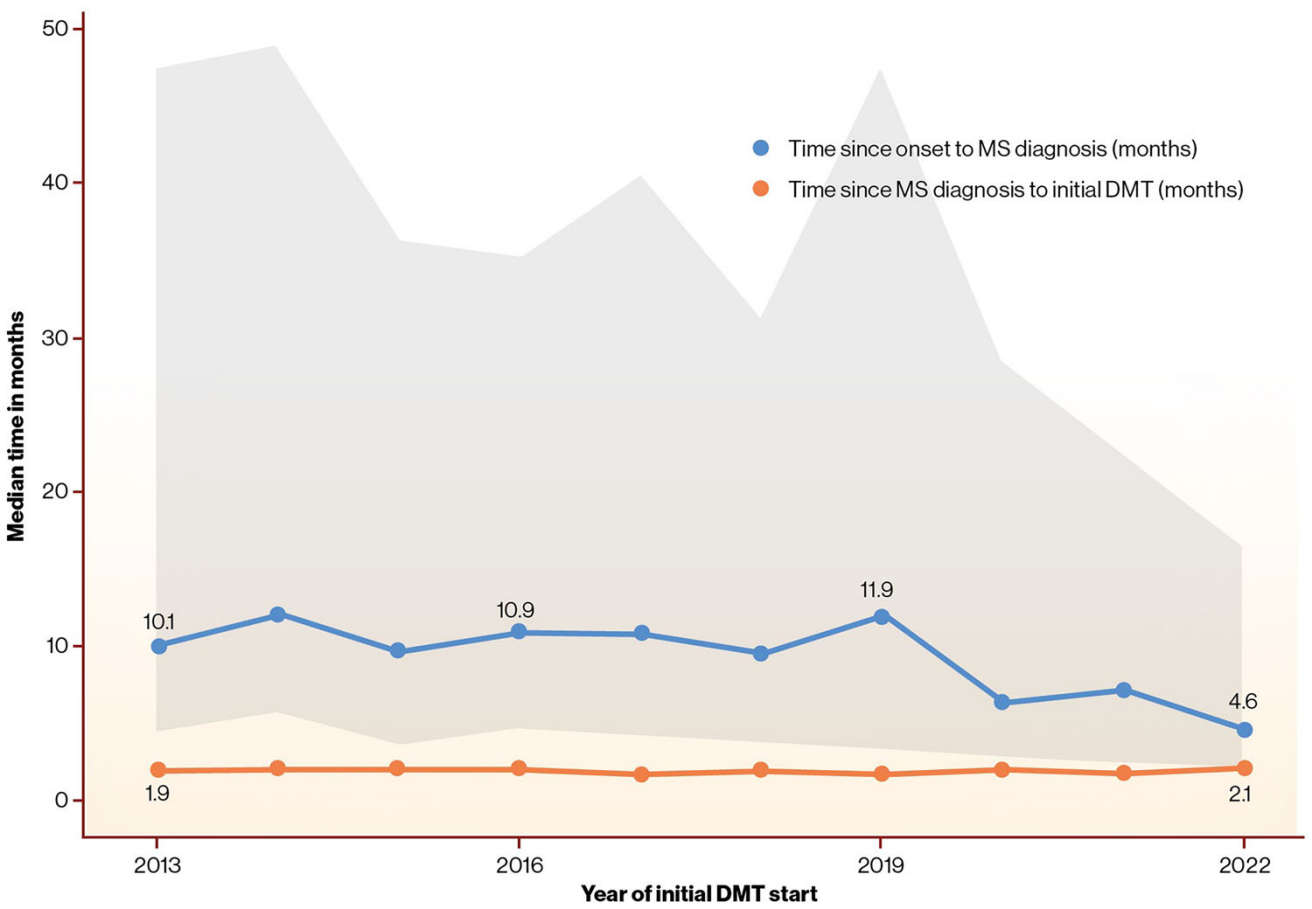
Evolution of time since onset to MS diagnosis and time since diagnosis to DMT initiation from 2013 to 2022. DMT, disease‐modifying therapy; MS, multiple sclerosis.

In 2020, there was a notable decrease in the diagnostic delay (Figure 3). The median diagnostic delay was 10.1 months in 2013 (IQR = 4.6–47.3), reducing to 4.6 months in 2022 (IQR = 2.1–16.4; *p* < 0.001). The time from MS diagnosis to the initiation of the first DMT remained consistent from 2013 to 2022, with a median of 1.9 months (IQR = 1.1–3.3).

Due to the significant decrease in diagnostic delay since 2020, the year of diagnosis was identified as the confounding factor for time delays from the first presentation of symptoms to diagnosis and to treatment initiation and the number of relapses.

The decrease in diagnostic delay since 2020 was significant in patients who received meDMTs as the initial DMT (Table 2). From 2013 to 2019, the median diagnostic delay was significantly longer in the meDMT group, compared with the heDMT group (5.2 months [IQR = 2.2–17.3]; *p* < 0.001). In 2020–2022, the median diagnostic delay in the meDMT group decreased notably from 11.8 months (IQR = 4.6–45.5) to 6.2 months (IQR = 2.6–27.9; *p* < 0.001).

**TABLE 2:** The demographics of patients with RRMS receiving their initial DMT between 2013 and 2022 in Finland by initial DMT year.

	Initial DMT year 2013–2019			Initial DMT year 2020–2022		
	meINJs + meORALs	heDMTs	*p* value^a^, ^b^	meINJs + meORALs	heDMTs	*p* value^a^, ^b^
Sex						
Female; *n* (%)	1134 (72.7%)	177 (71.4%)	0.65	289 (74.5%)	162 (69.8%)	0.24
Age variables (years); mean (SD)						
Age at MS onset	32.9 (9.58)	31.1 (8.96)	0.009^**^	33.3 (9.15)	32.2 (8.82)	0.19
Age at MS diagnosis	36.0 (9.77)	33.2 (9.47)	< 0.001^***^	36.4 (9.07)	34.5 (9.02)	0.02^*^
Age at the start of the initial DMT	36.4 (9.78)	33.5 (9.45)	< 0.001^***^	36.9 (9.03)	34.9 (9.08)	0.02^*^
Time delay variables (months); median [IQR]						
Time since MS onset to MS diagnosis	11.8 [4.6–45.5]	5.2 [2.2–17.3]	< 0.001^***^	6.2 [2.6–27.9]	6.2 [2.0–20.6]	0.20
Time since MS onset to the start of the initial DMT	16.0 [6.7–55.0]	8.0 [4.1–21.5]	< 0.001^***^	9.5 [4.8–32.1]	9.5 [4.0–24.3]	0.20
Time since MS diagnosis to the start of the initial DMT	1.9 [1.1–3.4]	1.8 [1.0–2.9]	0.14	1.8 [1.1–3.1]	2.0 [1.3–3.2]	0.24
Relapse variables; mean (SD)						
ARR 1 year before MS diagnosis	1.0 (0.70)	1.2 (0.84)	< 0.001^***^	0.7 (0.63)	1.1 (0.82)	< 0.001^***^
ARR 1 year before first DMT	0.9 (0.76)	1.3 (0.88)	< 0.001^***^	0.7 (0.65)	1.1 (0.82)	< 0.001^***^
EDSS score at the start of the initial DMT (0–10); median [IQR]	1.5 [1.0–2.0]	2.0 [1.2–3.0]	< 0.001^***^	1.5 [0.0–2.0]	2.0 [1.0–2.5]	< 0.001^***^
Onset symptom level^c^; *n* (%)						
Brainstem	237 (17.3%)	44 (20.6%)	0.28	54 (19.4%)	35 (19.1%)	1.00
Cerebellum	242 (17.6%)	52 (24.3%)	0.03^*^	34 (12.2%)	40 (21.9%)	0.02^*^
Optic nerve	350 (25.5%)	29 (13.6%)	< 0.001^***^	86 (30.8%)	25 (13.7%)	< 0.001^***^
Pyramidal tract	208 (15.2%)	55 (25.7%)	< 0.001^***^	40 (14.3%)	43 (23.5%)	0.03^*^
Sensory pathways	660 (48.1%)	112 (52.3%)	0.29	115 (41.2%)	92 (50.3%)	0.10
Spinal cord	30 (2.2%)	11 (5.1%)	0.03^*^	5 (1.8%)	8 (4.4%)	0.20
Unknown	187 (12.0%)	34 (13.7%)	—	109 (28.1%)	49 (21.1%)	—
Multifocal symptoms: yes; *n* (%)	111 (8.1%)	27 (12.6%)	0.05^*^	21 (7.5%)	24 (13.1%)	0.10

*Note*: meINJs (beta interferons and glatiramer acetate), meORALs (dimethyl fumarate, diroximel fumarate, and teriflunomide), heDMTs (alemtuzumab, cladribine, daclizumab, natalizumab, ocrelizumab, ofatumumab, and rituximab), and S1Ps (fingolimod and ponesimod). Brainstem (double vision, specific brain nerve symptoms, and other cranial nerve symptoms), cerebellum (disturbances of coordination and ataxia, vertigo, and disturbances of balance and posture), optic nerve (optic neuritis), pyramidal tract (muscle weakness and spasticity), sensory pathways (paresthesia/dysesthesia), spinal cord (bladder and bowel dysfunction).

Abbreviations: ARR, annualized relapse rate; DMT, disease‐modifying therapy; EDSS, Expanded Disability Status Scale; heDMTs, high‐efficacy disease‐modifying therapies; IQR, interquartile range; meINJs, medium‐efficacy injectables; meORALs, medium‐efficacy orals; MS, multiple sclerosis.

^a^ *p* values are calculated comparing heDMT patients to DMT groups meINJ and meORAL.

^b^ *p* values are corrected using Benjamini–Hochberg procedure to control the false discovery rate.

^c^ Individual patients can have multiple onset symptoms.

* *p* < 0.05.

** *p *< 0.01.

*** *p* < 0.001.

#### 3.2.3 Disease Activity

Higher disease activity in terms of EDSS score and ARR in the preceding year was observed in patients who received heDMTs as the initial DMT compared to patients initiated with a meDMT. The median EDSS score in the heDMT group was significantly higher at the time of DMT initiation compared with the meDMT group (2.0 vs. 1.0; *p* < 0.001). ARR was higher in the heDMT group compared with the meDMT group (1.2 vs. 0.9; *p* < 0.001).

ARR in the preceding year before the diagnosis or initial DMT was significantly lower in 2020–2022 compared with 2013–2019 in both meDMT and heDMT groups (Table 2).

#### 3.2.4 Symptoms at Onset

The most commonly, onset symptoms were affecting sensory pathways (47.7%), optic nerve (23.9%), and brainstem (18.0%). Compared with meDMTs, initiation with a heDMT was more likely in patients experiencing cerebellar (*p* = 0.005), pyramidal (*p* < 0.001), spinal cord (*p* < 0.001), or multifocal symptoms (*p* = 0.005) as the first MS symptoms. Patients with optic neuritis were more likely to be initiated on meDMT than on heDMT (*p* < 0.001).

In 2020–2022, the associations between DMT initiation and MS symptoms were similar than in 2013–2022, except for spinal cord and multifocal symptoms not being significantly associated with heDMT initiation anymore.

#### 3.2.5 Factors Associated With Initial DMT Selection

We conducted univariate analyses to evaluate the individual associations of sex, time delays, disease activity, and time periods of 2013–2019 or 2020–2022 with the initial DMT choice (meDMT or heDMT). The meDMT group was used as the reference group. Subsequently, a multivariate logistic regression model was applied to assess the combined associations of the variables and control for potential confounders (Table 3). In the final multivariate model, younger age, higher ARR, higher EDSS score, and the 2020–2022 time period were associated with higher probability of initiating a heDMT instead of a meDMT.

**TABLE 3:** Odds ratios for choosing high‐efficacy DMT as the first DMT.

	All patients OR (95% CI); *p* value	DMT start 2013–2019 OR (95% CI); *p* value	DMT start 2020–2022 OR (95% CI); *p* value
Univariate model			
Sex (female)	0.89 (0.71–1.11); 0.28	0.93 (0.70–1.26); 0.65	0.79 (0.55–1.14); 0.21
Age at initial DMT start (years)	0.97 (0.96–0.98); < 0.001^***^	0.97 (0.95–0.98); < 0.001^***^	0.98 (0.96–0.99); 0.009^**^
Time since onset to initial DMT start	0.93 (0.91–0.96); < 0.001^***^	0.92 (0.88–0.96); < 0.001^***^	0.96 (0.92–1.00); 0.08
ARR before initial DMT start	1.59 (1.40–1.80); < 0.001^***^	1.69 (1.44–1.99); < 0.001^***^	2.06 (1.64–2.61); < 0.001^***^
EDSS at initial DMT start	1.44 (1.31–1.58); < 0.001^***^	1.47 (1.29–1.67); < 0.001^***^	1.42 (1.22–1.65); < 0.001^***^
Year of initial DMT start (2020–2022)	3.76 (3.04–4.64); < 0.001^***^		
Multivariate model			
Sex (female)	0.86 (0.62–1.22); 0.40	0.93 (0.60–1.46); 0.75	0.80 (0.47–1.37); 0.41
Age at initial DMT start (years)	0.96 (0.94–0.98); < 0.001^***^	0.95 (0.93–0.98); < 0.001^***^	0.96 (0.93–0.99); 0.010^**^
Time since onset to initial DMT start	0.97 (0.93–1.01); 0.10	0.96 (0.90–1.01); 0.12	0.98 (0.92–1.04); 0.56
ARR before initial DMT start	1.78 (1.45–2.18); < 0.001^***^	1.47 (1.15–1.90); 0.003^**^	2.46 (1.74–3.56); < 0.001^***^
EDSS at initial DMT start	1.60 (1.42–1.80); < 0.001^***^	1.60 (1.38–1.86); < 0.001^***^	1.63 (1.34–2.00); < 0.001^***^
Year of initial DMT start (2020–2022)	3.42 (2.52–4.66); < 0.001^***^		

Abbreviations: ARR, annualized relapse rate; CI, confidence interval; DMT, disease‐modifying therapy; EDSS, Expanded Disability Status Scale; OR, odds ratio.

** *p* < 0.01.

*** *p* < 0.001.

### 3.3 Initial DMT Discontinuation and Switch

#### 3.3.1 Initial DMT Persistence and Reasons for Discontinuation During the First 2 Years From 2013 to 2020

Within the first 2 years, the initial DMT was discontinued in 52.0% of patients with meINJs, in 31.7% with meORALs, and in 27.5% with heDMTs (Table 4). Among patients initiated on meDMTs, the most frequent reason for discontinuation was adverse effects (meINJs 53.6% and meORALs 55.1%). Among patients initiated on heDMTs, the most frequent reason for discontinuation was John Cunningham (JC) virus (43.2%).

**TABLE 4:** The reasons for initial DMT termination during the first 2 years in patients with RRMS in Finland receiving their first DMT between 2013 and 2020.

	All patients (*N* = 2115)	meINJs (*n* = 942)	meORALs (*n* = 788)	heDMTs (*n* = 338)	S1PRms (*n* = 42)
Initial DMT terminated during the first 2 years; *n* (%)	845 (40.0%)	490 (52.0%)	250 (31.7%)	93 (27.5%)	9 (21.4%)
Reasons for DMT termination^a^; %					
Adverse effects	49.4%	53.6%	55.1%	13.6%	22.2%
Alteration of disease course	< 1%	1.1%	0.0%	2.5%	0.0%
Clinically ineffective	23.2%	20.9%	30.7%	11.1%	55.6%
Drug antibodies	4.5%	6.6%	< 1%	3.7%	0.0%
JC virus	4.5%	0.0%	0.0%	43.2%	0.0%
Patient's wish	9.8%	11.5%	7.1%	7.4%	11.1%
Pregnancy	7.6%	9.4%	4.4%	7.4%	0.0%
Other	5.6%	3.6%	5.8%	13.6%	11.1%
Reason unknown	7.0%	4.5%	10.1%	12.9%	0.0%

*Note*: meINJs (beta interferons and glatiramer acetate), meORALs (dimethyl fumarate, diroximel fumarate, and teriflunomide), heDMTs (alemtuzumab, cladribine, daclizumab, natalizumab, ocrelizumab, ofatumumab, and rituximab), and S1PRms (fingolimod and ponesimod).

Abbreviations: DMT, disease‐modifying therapy; heDMTs, high‐efficacy disease‐modifying therapies; JC, John Cunningham; meINJs, medium‐efficacy injectables; meORALs, medium‐efficacy oral therapies; RRMS, relapsing‐remitting multiple sclerosis; S1PRms, sphingosine 1‐phosphate modulators.

^a^ Individual patients can have multiple reasons for DMT termination.

#### 3.3.2 Lack of Efficacy

A subpopulation analysis was conducted in patients who had their initial DMT discontinued at any point between 2013 and 2022 due to insufficient efficacy (*n* = 301). In this subcohort, slightly higher proportion (75.7%) were female than in the whole study population. The mean age at the time of second DMT initiation was 35.8 years (SD = 9.07). The median time on initial DMT was 1.3 years (IQR = 0.8–2.7). The median time from the disease onset to the second DMT was 3.3 years (IQR = 1.8–6.0). The most common second DMTs were heDMTs (44.2%), followed by S1PRms (32.9%), meORALs (16.3%), and meINJs (4.3%). No second DMT was initiated for 2.0% of patients.

### 3.4 Switch Patterns

**FIGURE 4:**
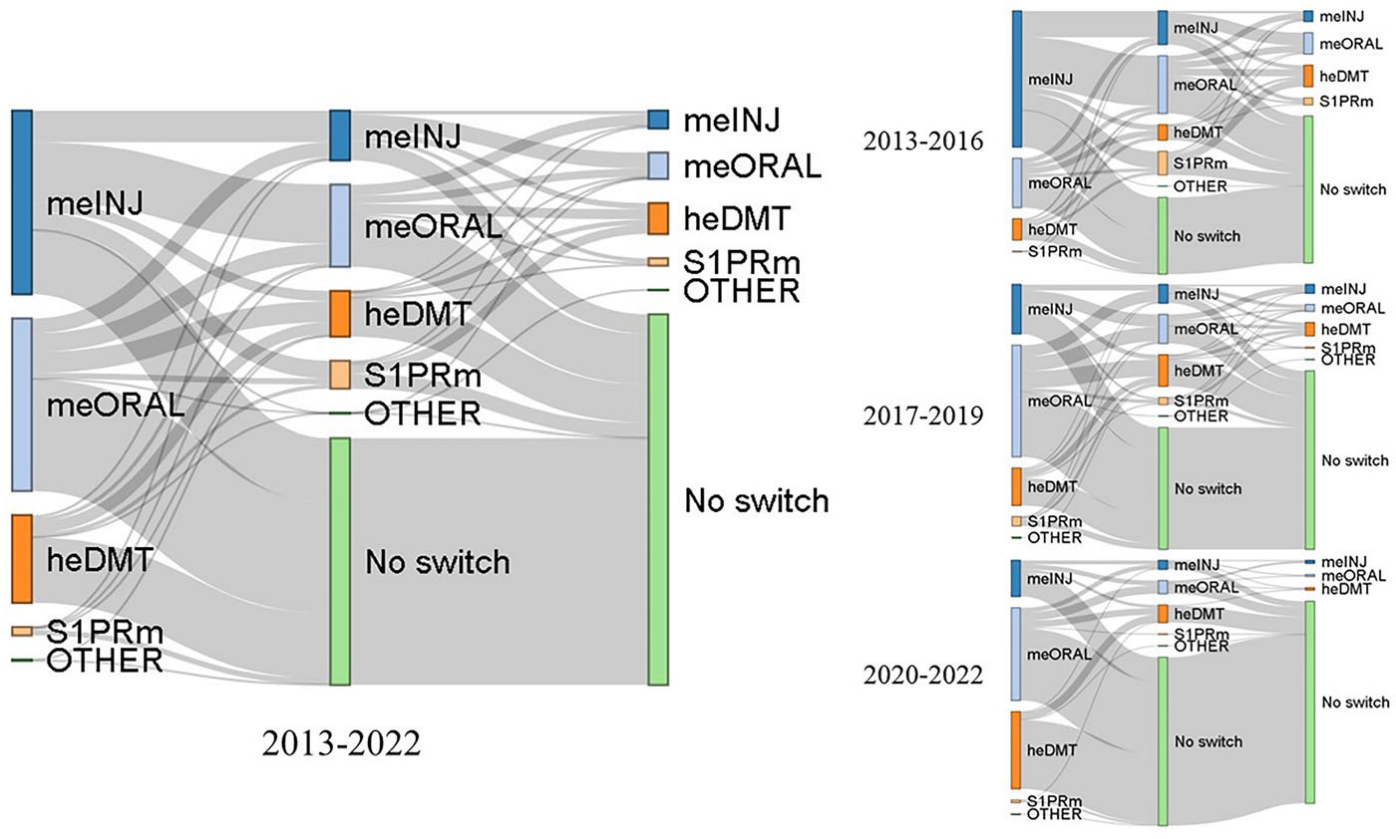
Switch patterns of the first and the second DMT switch. DMT, disease‐modifying therapy; heDMT, high‐efficacy disease‐modifying therapy; meINJ, medium‐efficacy injectable; meORAL, medium‐efficacy oral; S1PRm, sphingosine 1‐phosphate receptor modulator.

While most switches (69% of all the switches from 2013 to 2022) from meDMTs were made to a DMT of similar efficacy, an increasing frequency of switches toward more efficient DMTs was observed over time (Figure 4).

## 4 Discussion

The percentage of heDMTs as the initial DMTs has significantly increased in Finland in the recent years, being nearly 44% in 2022. Apparently, the treatment paradigm is shifting from an escalation strategy toward EIT. Similar numbers have been reported in Sweden (35%–42% heDMTs of the initial DMTs; Hrnciarova et al. 2023; Spelman et al. 2021), and there has been a growing trend of initial heDMTs in Denmark (Buron et al. 2020), whereas the traditional escalation strategy has been used, for example, in Czech Republic (3.8%) (Hrnciarova et al. 2023). Country‐wise heterogeneity in care practices likely reflects the lack of consensus of the optimal treatment strategy in RRMS. Currently, neither European (Montalban et al. 2018) nor American (Rae‐Grant et al. 2018) guidelines recommend any specific treatment strategy.

It is likely that EIT is more effective than the escalation strategy in terms of MS prognosis (Hrnciarova et al. 2023; Spelman et al. 2021; Buron et al. 2020; Hänninen et al. 2022; Harding et al. 2019) The rationale for using the escalation strategy is that the effectiveness of heDMTs comes at the cost of increased risk of rare yet potentially serious side effects. For example, the European Medicines Agency has recommended limiting the use of alemtuzumab (European Medicines Agency 2019) due to its association with a higher risk for immune‐mediated diseases and serious cardiovascular events (Coles et al. 2017). The currently used heDMTs have demonstrated being relatively safe in short‐term use with appropriate follow‐up. The adverse events of anti‐CD20 DMTs (ocrelizumab [Hauser, Bar‐Or, Comi et al. 2017], ofatumumab [Hauser, Bar‐Or, Cohen et al. 2020)], and rituximab [Kaegi et al. 2019]) are mainly associated with injection‐ or infusion‐related reactions and a slightly higher infection risk. Natalizumab has demonstrated an acceptable safety profile (Polman et al. 2006) except for the association with progressive multifocal leukoencephalopathy, the risk of which, however, can be significantly lowered by monitoring the patients for serum anti‐JC virus antibodies (Bloomgren et al. 2012). Cladribine is associated with the risk of lymphopenia and herpes zoster infection (Giovannoni et al. 2010). Long‐term safety data for heDMTs are limited owing to their novelty in MS treatment and thereby more research is needed. Eventually, choosing the treatment strategy is based on the benefit–risk ratio. In our data, patients who had to switch their initial meDMT due to the lack of efficacy experienced a median time of 3.3 years from the disease onset to the second DMT. In these patients, the escalation strategy means that the disease activity may be insufficiently controlled for over 3 years in the period known to be critical to long‐term disease outcomes (Lefort et al. 2022; Iaffaldano et al. 2021; Chalmer, Baggesen et al. 2018; Kavaliunas et al. 2017). This should be considered as a risk of the escalation strategy. The choice of medication is also influenced by several patient‐related factors, such as age, comorbidities, other medications, DMT‐specific issues, plans of pregnancy, and personal preferences. The patient should be involved in the decision‐making by highlighting all the advantages and disadvantages of each treatment strategy.

The choice of the treatment strategy has likely been influenced by Finnish drug‐specific reimbursement criteria. All the meDMTS available in Finland from 2013 to 2022 were fully reimbursed for the treatment of RRMS (except for diroximel fumarate until 2022). Fingolimod and cladribine were fully reimbursed only for patients with highly active disease, thus, limiting their use as the initial treatment choice. Ponesimod was not fully reimbursed. As natalizumab, ocrelizumab, and rituximab are administered in a hospital setting, the reimbursement system does not apply to them, but the resources of the hospital may limit the use of the drugs. Ofatumumab was fully reimbursed for the treatment of RRMS at the end of 2022.

The median diagnostic delay for all patients was 10.6 months from 2013 to 2019 and 6.2 months from 2020 to 2022. A similar trend toward shorter diagnostic delay has been observed in other countries as well (Magyari et al. 2022; Blaschke et al. 2022; SMSREG 2024). The reduction is most likely due to the updated McDonald diagnostic criteria in 2017 (Thompson et al. 2018), having greater sensitivity and thereby resulting in shorter diagnostic delay compared with the earlier 2010 criteria (Filippi et al. 2022). The publication of the Finnish current care guidelines for MS with McDonald 2017 criteria in 2020 (The Finnish Medical Society Duodecim and the Finnish Neurological Society 2020) is likely the cause for the observed decline in the diagnostic delay from 2020 onward. Shorter treatment delay has been demonstrated to reduce the risk of disease progression (Lefort et al. 2022; Iaffaldano et al. 2021; Chalmer, Baggesen et al. 2018; Kavaliunas et al. 2017). Our data suggest a decreasing trend in diagnostic delay most evidently in patients who were first initiated on meDMT. These patients have presumably benefited the most from the application of the new diagnostic criteria. According to our regression analysis, initiation with meDMT was associated with older age and lower disease activity. The decrease in diagnostic delay is probably the reason for the observation that ARR preceding the diagnosis was significantly lower in both heDMT and meDMT groups after 2020. The COVID‐19 pandemic did not increase the diagnosis or treatment delays, contrary to our hypothesis. Although the pandemic caused difficulties in providing health care services, it was not as extensive in Finland as in many other countries (Tiirinki et al. 2020).

According to our regression analysis, patients with higher disease activity at the time of DMT initiation, in terms of EDSS score and ARR, were more likely to have heDMT as the initial DMT. Moreover, initial symptoms clinically originating from cerebellum, spinal cord, or pyramidal tract, as well as symptoms of multifocal origin, were associated with heDMT initiation. These symptoms may reflect higher disease activity at the disease onset or greater clinical concern associated with certain symptoms. On the contrary, optic neuritis, related to the initiation of meDMT, might be associated with less concern. Previous studies have shown mixed results of different symptoms at onset on prognosis, except for bowel or bladder involvement, which have been associated with worse prognosis in most studies (Langer‐Gould et al. 2006). From 2020 to 2022, multifocal symptoms and spinal cord symptoms were not significantly associated with the initiation of either DMT group. This might be due to the changing treatment paradigm, as heDMTs were more frequently initiated in patients with lower disease activity. The smaller sample size in 2020–2022 could also affect the accuracy of the results.

DMT persistence during the first 2 years was the lowest among patients receiving meDMTs, adverse effects being the most common reason for discontinuation. The heDMTs were generally tolerated better. This is in line with the previous studies, which have demonstrated that heDMTs have lower dropout rates than meDMTs (Li et al. 2020). However, we cannot draw conclusions about the safety of the DMTs, as we did not assess the severity of the adverse effects.

Patients who discontinued DMTs due to ineffectiveness primarily switched to higher‐efficacy DMTs. Switching to heDMTs became increasingly common from 2013 to 2022. The first meDMT switch due to any reason was most often a switch to a DMT of similar efficacy. Previous studies have found switching from meDMTs to heDMTs results in better disease outcomes (Guger et al. 2023; Chalmer, Kalincik et al. 2019). Adverse effects, being the most common reason for discontinuing a meDMT, are probably the major reason for switching from an initial meDMT to another. Oral therapies appear to be preferred over meINJs as meORALs were a more frequent choice for the second treatment. A previous Finnish study showed that DMT switches from injectable therapies to orals peaked in 2015 corresponding with teriflunomide becoming available in 2014 and dimethyl fumarate in 2015 (Lahdenperä et al. 2020).

This study has several strengths. Extensive multicenter register data were used in this study, with national coverage of about 85% of patients with MS, which makes the results of this study generalizable in Finland. Clinical relapses, time of diagnosis, time of first symptoms, and the reasons for the medication switches were all comprehensively documented in the register. The risk of selection bias was low as we included all the patients with RRMS diagnosis and on initial DMTs.

We recognize several limitations of the study. We did not have fixed follow‐up periods, which limited longitudinal analyses and was a potential source of time‐dependent bias regarding DMT switch patterns, which may have caused overrepresentation of quickly switched DMTs toward the end of the follow‐up period. Thus, comparing the time periods of the switch patterns should be approached with caution. A longer follow‐up period would provide the opportunity to assess in later stages of RRMS and would enable examining the effect of disease activity on treatment decisions, which would be an interesting topic for further research. MRI data were insufficient for the analyses as all the MS register centers did not collect the MRI data systematically. We recommend future studies to incorporate MRI data as it provides an additional perspective for evaluating disease activity. Finally, as in all register studies, evaluating the quality of the data obtained from the register is difficult.

In summary, RRMS care in Finland has shifted toward earlier diagnosis and earlier initiation of heDMTs. Initiation with a heDMT was associated with younger age and higher disease activity. Switching from meDMTs to heDMTs is increasingly prevalent among patients discontinuing DMTs due to insufficient treatment efficacy. Finally, the Finnish health care system was able to adapt to the COVID‐19 pandemic in terms of early diagnosis and treatment of patients with RRMS.

## Author Contributions

**Henrik Ahvenjärvi**: writing–original draft, visualization, conceptualization, investigation, funding acquisition. **Elina Jokinen**: project administration, funding acquisition, writing–review and editing, conceptualization. **Matias Viitala**: formal analysis, visualization, methodology, writing–original draft, conceptualization, software, validation, investigation, data curation. **Henri Autio**: conceptualization, funding acquisition, writing–review and editing, project administration. **Anne M. Portaankorva**: supervision, writing–review and editing. **Merja Soilu‐Hänninen**: writing–review and editing, conceptualization. **Johanna Krüger**: writing–review and editing, conceptualization, supervision. **Mervi Ryytty**: conceptualization, writing–review and editing, supervision, funding acquisition.

## Conflicts of Interest

Henrik Ahvenjärvi has received research grants from the Finnish Neurological Society, the Finnish Cultural Foundation, the Finnish MS Foundation, and the University of Oulu Scholarship Foundation and support for conference and education session participation and honoraria for consultant services from Novartis. Elina Jokinen is an employee of Novartis Finland Ltd. Henri Autio is a former employee of Novartis Ltd. Matias Viitala is an employee of StellarQ Ltd. Merja Soilu‐Hänninen has served as an adviser or speaker for Biogen, Novartis, Roche, Sanofi, and Teva and received support for congress participation from Biogen, Merck, and Novartis. Johanna Krüger has served on scientific advisory boards, received support for congress participation, served as a consultant, and received speaker honoraria from Eisai, Bioarctic, Merck, Novartis, Roche, and Roche Diagnostics. Anne M. Portaankorva reports no conflicts of interest. Mervi Ryytty has received honoraria for lectures, advisory boards, congress visit, and for serving as an investigator for clinical trials from AbbVie, Biogen, Merck, Novartis, Roche, Sandoz, and Sanofi.

### Peer Review

The peer review history for this article is available at https://publons.com/publon/10.1002/brb3.70326.

## Data Availability

The research data cannot be shared due to restrictions caused by Finnish and European Union legislation.
